# Incidental Finding of Ante-natal Fetal Neck Mass With Post-delivery Imaging and Follow-Up

**DOI:** 10.7759/cureus.59463

**Published:** 2024-05-01

**Authors:** Sheetal S Shelar, Pratap Parihar, Shirish Vaidya, Rajasbala Dhande, Asish Pavanan

**Affiliations:** 1 Radiodiagnosis, Jawaharlal Nehru Medical College, Datta Meghe Institute of Higher Education and Research, Wardha, IND

**Keywords:** thyroid, computed tomography, goitre, neck masses, ultrasound, fetal anomalies

## Abstract

Most fetal anomalies can be detected during the second trimester of chromosomal anomaly screening. However, even an experienced sonographer might fail to notice a fetal neck mass during this screening and would be diagnosed at a later point in time. In this case report, we have followed up on an incidentally detected case of fetal neck mass on antenatal sonography with post-delivery ultrasound and contrast-enhanced computed tomography.

## Introduction

The occurrence of fetal neck masses is rare, and ultrasonography is the primary modality for antenatal detection [1]. Most of these masses are benign, cystic hygroma being the most common [2,3]. Other masses, such as hemangioma, goiter, and cervical teratoma, are less frequently encountered [4-6]. Detection of these masses prenatally by ultrasound or MRI may give an accurate diagnosis namely the location and extension [7]. Some of these masses can be managed by in-utero treatment, while a few may be associated with aneuploidies and congenital syndromes [8]. Therefore, prenatal scanning can help in the selection of patients who require early treatment.

## Case presentation

A 34-year-old woman (gravida 7, para 1, living 1, abortions 5) (all the abortions occurring at one month of pregnancy spontaneously) came to ANC OPD at 33.5 weeks of gestation by LMP (last menstrual period) for a routine ANC scan to evaluate fetal growth, weight, and parameters with color flow mapping and duplex doppler study. The woman has a history of hypothyroidism (Hashimoto's thyroiditis was detected in the third month of the previous pregnancy and is currently on medications for the same). Even then, her TSH values were found to be significantly raised compared to average values in pregnancy. She was also diagnosed with hypertension and gestational diabetes mellitus in the first month of the previous pregnancy and is taking medications for the same. However, the fetal growth parameters like biparietal diameter (BPD), head circumference (HC), femur length (FL), and abdominal circumference (AC) were normal. The liquor index and Doppler study values were also within normal limits.

**Figure 1:**
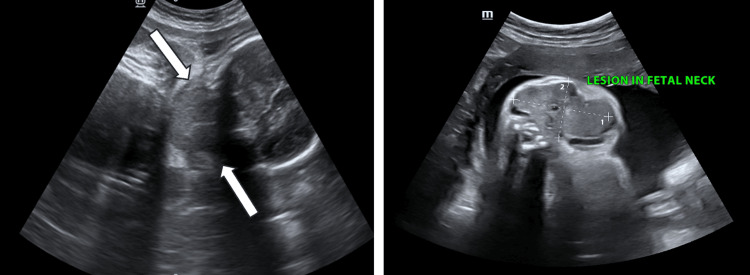
(Left and right) A grey-scale ultrasound image of fetal neck showing the bilaterally symmetrical cervical mass.

**Figure 2:**
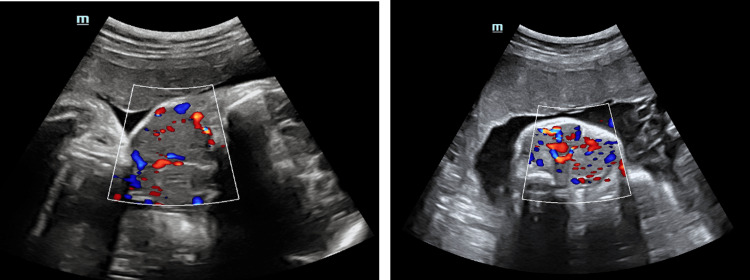
(Left and right) Color Doppler images showing vascularity within the fetal neck mass.

On ultrasonography, a well-defined uniformly iso-echoic, solid, and bilaterally symmetrical mass was observed in the anterior neck, extending from the supra-clavicular region up to the base of the skull (Figure 1). On color Doppler, the mass showed the presence of moderate internal vascularity (Figure 2).

**Figure 3:**
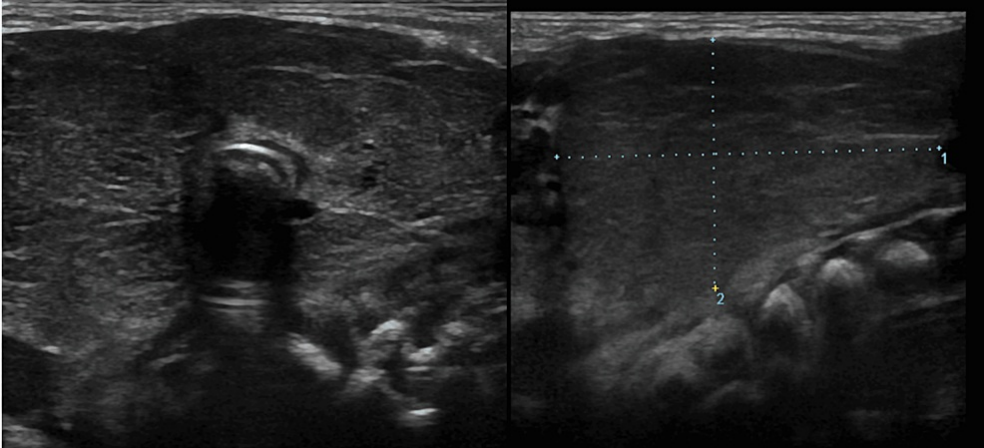
(Left and right) A grey-scale ultrasound image of diffusely enlarged thyroid gland.

At 37 weeks, the caesarian section was performed in view of early rupture of membranes. Ultrasonography of the neck mass was done with a linear probe which showed evidence of enlargement of bilateral lobes of the thyroid gland (Figure 3).

**Figure 4:**
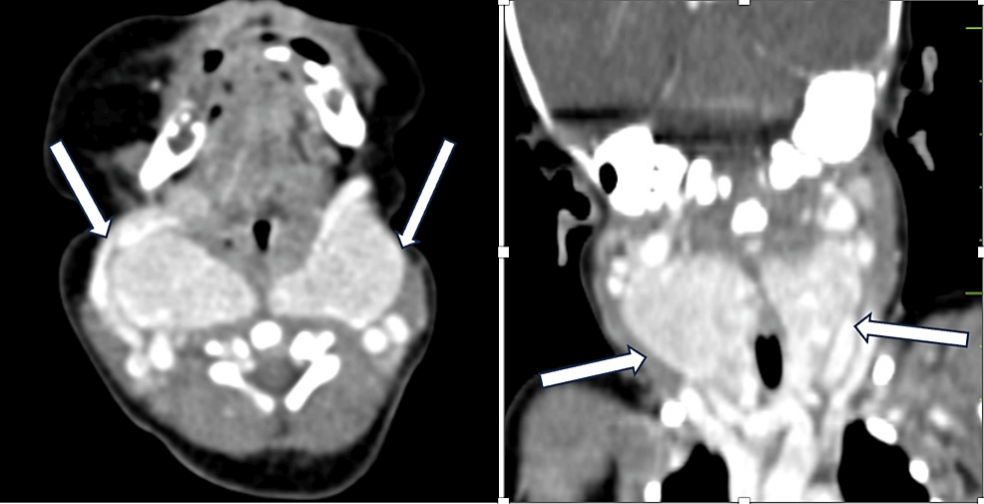
Axial and coronal CECT images of neonatal neck showing intensely enhancing enlarged thyroid gland with few cystic areas within.

The neck mass was further investigated with routine blood tests including a thyroid profile. TSH and T4 levels were within normal limits with slightly decreased T3 levels. Serum iodine levels and the rest of the blood parameters were also normal. Further, contrast-enhanced computed tomography was done which revealed a well-defined intensely enhancing diffusely enlarged bilateral lobes of thyroid and isthmus with few internal cystic areas. The enlarged gland is extending from the C2 to the supra sternal notch causing a mass effect in the form of displacement of the right external and internal carotid arteries and left external carotid artery laterally with no evidence of retrosternal extension (Figure 4).

## Discussion

Congenital neck masses are occasionally encountered in routine antenatal screening and are frequently missed compared to other congenital anomalies. Nonetheless, ultrasonography has been the modality of choice for early detection and serial follow-up. They can be divided based on their location into anterior and posterior cervical masses. Also, the nature of the mass whether solid or cystic, internal solid component and vascularity, presence of septations or calcifications should be taken into account [9]. Benign masses are relatively commoner, goiter being one of them. Differentiating various pathologies is important for proper management and mode of action. The cervical tumor can cause mass effect in the form of compression of the esophagus in-utero impairing fetal swallowing leading to polyhydramnios and preterm delivery or the trachea causing airway obstruction, subsequent hypoxia, and death [7,10]. Hence, post-delivery imaging is necessary for doubtful diagnoses or accurate location and extension of the neck mass.

## Conclusions

Detection of fetal neck masses in early trimesters to plan the mode of delivery and postnatal management is as important as diagnosing other congenital anomalies. Cervical tumors are most likely benign. However, they are difficult to identify on antenatal ultrasonographic examination. Serial follow-up is required in order to narrow down the diagnoses and for subsequent treatment.
